# Exploring Mast Cell–CD8 T Cell Interactions in Inflammatory Skin Diseases

**DOI:** 10.3390/ijms24021564

**Published:** 2023-01-13

**Authors:** Yiqiao Chen, Christopher E. M. Griffiths, Silvia Bulfone-Paus

**Affiliations:** Lydia Becker Institute of Immunology and Inflammation, Dermatology Research Centre, NIHR Manchester Biomedical Research Centre, University of Manchester, Manchester M13 9PL, UK

**Keywords:** mast cells, CD8 T cells, bidirectional interaction, signaling pathway, costimulatory molecule

## Abstract

The skin is exposed to environmental challenges and contains skin-resident immune cells, including mast cells (MCs) and CD8 T cells that act as sentinels for pathogens and environmental antigens. Human skin MCs and their mediators participate in the maintenance of tissue homeostasis and regulate the recruitment and activity of immune cells involved in the pathogenesis of skin diseases. The cutaneous CD8 T cell compartment is comprised of long-persisting resident memory T cells (T_RM_) and migratory or recirculating cells; both populations provide durable site immune surveillance. Several lines of evidence indicate that MC-derived products, such as CCL5 and TNF-α, modulate the migration and function of CD8 T cells. Conversely, activated CD8 T cells induce the upregulation of MC costimulatory molecules. Moreover, the close apposition of MCs and CD8 T cells has been recently identified in the skin of several dermatoses, such as alopecia areata. This review outlines the current knowledge about bidirectional interactions between human MCs and CD8 T cells, analyses the alteration of their communication in the context of three common skin disorders in which these cells have been found altered in number or function—psoriasis, atopic dermatitis, and vitiligo—and discusses the current unanswered questions.

## 1. Introduction

Skin forms an effective first-line barrier against external environmental hazards [1]. The cutaneous physical, chemical, microbiological, and immune systems function in a coordinated manner to respond rapidly and effectively to a wide variety of insults in order to maintain skin homeostasis [2]. According to the Global Burden of Disease (GBD) project, dermatological diseases are ranked fourth by the incidence of all causes of disease, affecting approximately one-third of the global population [3,4]. Major skin-related autoimmune diseases, such as pemphigus, alopecia areata (AA), atopic dermatitis (AD), rosacea, vitiligo, and psoriasis are characterised by abnormal immune responses mounted against skin-specific or ubiquitous self-antigens [5]. These responses result in local and/or systemic damage, dysregulated barrier (keratinocyte) function, and vasculopathy [6]. A large body of evidence from immunobiological screening and landscape mapping of the microenvironment of diseased skin has identified aberrant skin-resident immune cells as a mutual characteristic of various dermatoses, with mast cells (MCs) and CD8 T cells playing a pivotal role [7,8,9]. MCs function as effector cells in innate immunity as well as immunoregulatory cells in adaptive immunity, while CD8 T cells are the major effector cells of the adaptive immune system to orchestrate antigen-specific skin immune responses against pathogens [10,11]. The complex cellular networks between adaptive and innate immune cells are crucial for maintaining skin homeostasis [12]. Recent large-scale genome-wide association studies (GWAS) have revealed a remarkable overlap of epigenetic factors between AD and psoriasis, strengthening the concept of a shared inflammation-associated signature that is of general importance for various immune-mediated inflammatory skin diseases [13]. This highlights the importance of understanding shared downstream pathways and intercellular reactions in exacerbating disease conditions and developing novel therapeutic strategies [14]. This review summarises current knowledge of the mechanisms of interactions between MCs and CD8 T cells, and it discusses the alteration of these communication pathways in representative MC/CD8 T cell-dysregulated skin diseases: vitiligo, psoriasis, and AD.

## 2. Mast Cells and CD8 T Cells in Tissue Homeostasis and Disease

### 2.1. Mast Cells

MCs are long-lived tissue-resident cells of myeloid lineage with newly discovered dual hematopoietic origins [11,15]. Committed progenitor cells enter the bloodstream and circulate to peripheral tissues (e.g., skin, lung, and intestine), where they differentiate and mature under the influence of the local microenvironment [15]. MCs exhibit a high degree of phenotypic and functional heterogeneity as well as plasticity, which likely derives from different MC developmental and differentiation programmes imprinted by tissue-specific microenvironments [16,17]. In normal human skin, MCs are identified mainly as tryptase- and chymase-expressing MC_TC_ concentrated in the dermis, especially in close proximity to nerve fibres, hair follicles, and vascular endothelial cells [18,19]. Cutaneous MCs are first-line sentinels against pathogen invasion, shaping innate and adaptive immunity, and promoting wound healing [20]. They are equipped with a variety of cell surface receptors (e.g., high-affinity IgE Fc receptor (FcεRI)) that can recognise antigen epitopes and initiate a rapid response to stimuli, including complement and opiates [21]. Antigenic crosslinking of the FcεRI and IgE complex is best known for triggering FcεRI-dependent MC activation in allergic processes [22,23], whereas recent studies have uncovered a novel FcεRI/IgE-independent MC activation pathway which relies on the engagement of the MAS-related G protein-coupled receptor-X2 (MRGPRX2) [24]. MRGPRX2 is expressed predominantly by skin-resident MCs, and its ligation elicits MC degranulation characterised by a distinct granule-releasing pattern and cytokine expression profile [25]. Upon degranulation, MCs rapidly release preformed inflammatory mediators, including growth factors (e.g., stem cell factor (SCF) and vascular endothelial growth factor (VEGF)), histamine, proteoglycans, proteases (e.g., tryptase and carboxipeptidase), and cytokines (e.g., TNF-α), followed by secretion of a plethora of de novo synthesised proinflammatory products, including lipid mediators (e.g., prostaglandin D2 (PGD2)), cytokines, and chemokines (e.g., IL-4 and IL-6) [26,27]. These MC-derived products are known to participate in epithelial proliferation and the selective recruitment, activation, and regulation of immune cells (e.g., neutrophils, eosinophils, dendritic cells, and T lymphocytes) [28]. 

MCs are proinflammatory in pathogenic circumstances but can act as both anti-inflammatory and immunosuppressive under physiological conditions [29]. In addition to contributing to allergic reactions, studies in human tissues and mouse models using histological approaches and single-cell transcriptome analysis have demonstrated that MCs are associated with a range of inflammatory skin diseases [30]. At sites of inflammation, they show an increased density, degranulation, and proliferation, along with distinct disease-associated cytokine production [31,32]. Bertolini et al. proposed that the increased number of MCs in AA results from enhanced local proliferation of MCs and increased recruitment of progenitor MCs from the circulation [33]. This hypothesis is supported by Keith and colleagues, who distinguished bone-marrow-derived MC (BMMCs) from resident MCs on the basis of integrin-β7 expression in murine models of AD [34,35]. In psoriasis, activated MCs expressing IL-17, TNF-α, and IL-22 are consistently found enriched in involved and uninvolved skin compared with normal skin [36]. In addition, the expression of MRGPRX2 is increased in chronic idiopathic urticaria and rosacea [37]. The switch of MC-phenotype from anti-inflammatory to proinflammatory, as shown by a decreased expression of IL-10 and TGF-β along with increased tryptase immunoreactivity, has been reported in skin diseases, such as AA and severe cutaneous contact hypersensitivity (CHS) [26,33]. 

In addition to the initiation and development of skin disorders, cutaneous MCs may participate in disease reoccurrence, which is shown to be associated with pathogenic immune cells in perilesional skin and/or their persistence in resolved skin [38]. In bullous pemphigoid, IgE- or BP180-expressing MCs were found in perilesional skin and degranulate in a FcεRI-dependent manner [39]. Despite the clinical regression of disease symptoms by effective conventional treatment, such as psoralen and ultraviolet light A (PUVA) or local corticosteroid, MC depletion was found incomplete in resolved psoriasis lesions [40]. Furthermore, MC degranulation and the subsequent appearance of recruited immune cells occurring after treatment may contribute to the re-emergence of plaques at the site of clinical clearance [41,42]. 

### 2.2. CD8 T Cells

When encountering antigens presented by antigen-presenting cells (APCs), e.g., dendritic cells (DCs), circulating naïve CD8 T cells acquire an effector phenotype, recognise infected or damaged cells in a major histocompatibility complex (MHC) I-dependent manner, and initiate cell-killing at the site of inflammation [43]. Following initial stimulation, a pool of effector CD8 T cells differentiates into memory cells to provide a long-term protective immunity [44]. In humans, memory CD8 T cells infiltrating the skin are classified as recirculating or resident with diverse migratory and functional properties [10,45] (Table 1). Resident memory T cells (T_RM_) cells are a non-circulating subset of memory T cells located in peripheral tissues and provide long-term protection against previously exposed pathogens [46,47,48,49]. In healthy human skin, the majority of T_RM_ cells are dermal CD4 T cells, while CD8 T_RM_ cells localize mainly in the epidermis [12,50,51]. T_RM_ cells underpin the immune surveillance as cytotoxic killer cells and rapidly express proinflammatory cytokines and chemokines after reinfection [52]. Depending on their cytokine expression profile, T_RM_ cells are classified as CD49a^-^ CD8 T_RM_ cells, expressing Th2 cytokines and IL-17, and CD49a^+^ CD8 T_RM_ cells, which localize mainly to the epidermis, expressing interferon (IFN)-γ and cytokines belonging to the IL-22 family and cytotoxicity-associated molecules, such as granzyme B and perforin, upon IL-15 stimulation [53,54]. Recirculating memory T cells, divided into effector memory T cells (T_EM_) and central memory T cells (T_CM_) on the basis of CCR7 expression levels, transiently recirculate through lymphoid organs in response to sphingosine-1-phosphate (S1PR1) and CCL21 gradients [55]. The importance of this cell type in skin host immunity was demonstrated by the impaired host defence against infection in the skin of vaccinia virus mice with deleted antigen-specific recirculating memory T cells [56]. The synergistic function of CD8 T_RM_ cells and recirculating CD8 T cells within tissues is model-specific and pathogen-specific, as discussed by Richmond et al. [45]. 

In addition to host immunity, T_RM_ and recirculating T cells contribute to various human autoimmune diseases by means of antigen identification and secretion of cytokines and chemokines [57,58]. In the vitiligo mouse model, melanocyte-specific autoreactive T_RM_ cells have been shown in skin lesions [59]. Research by Gunderson et al. suggests that CD8 T cells are capable of mediating psoriasis-like skin phenotypes, including keratinocyte hyperproliferation via the IFN-γ-mediated mitogen-activated protein kinase (MAPK) signalling pathway [60]. Activated by tissue-damage-induced extracellular nucleotides, CD8 T_RM_ can also express a high level of the purinergic receptor P2RX7, a damage/danger-associated molecular pattern (DAMP) that promotes the differentiation of pro-inflammatory Th17 lymphocytes and enhances CD8 T_RM_ cell-sensing of TGF-β, which, in turn, supports the cell persistence in skin (Table 1) [61,62,63,64]. Compared with T_RM_ cells that are best investigated in skin disorders, the function of recirculating memory CD8 T cells in skin disorders has not been fully elucidated. 

Similar to MCs, CD8 T cells have been reported to play a role in the reoccurrence of skin disease. In vitiligo, a considerable proportion of CD8 T_RM_ persists in the perilesional skin of patients with stable disease, especially in the area where melanocytes are disappearing [39,65,66]. Notably, these cells preserve cytotoxic function with the expression of pro-inflammatory TNF-α and IFN-γ upon melanocyte antigen-specific activation [59]. Although lesions may occur in new areas, it is well known that recurrence often appears at previously healed sites, suggesting the concept of immune memory [67]. In allergic contact dermatitis, the number of epidermal CD8 T_RM_ cells was found to correlate with the intensity of disease flare-up reactions and are responsible for massive infiltration of neutrophils to the sites with chemokine-induced allergen rechallenge [68]. Similarly, a small population of CD8 T cells expressing cutaneous lymphocyte-associated antigen (CLA), CCR6, CD103, and IL-23R remains in clinically resolved plaques of psoriasis [69]. Locally maintained T_RM_ cells are capable of initiating in situ inflammation characterised by IL-17 production when interacting with antigens presented by DCs [70,71,72]. This corresponds to the persisting upregulation of several disease-related inflammatory genes (IL-17, IL-22, and IFN-γ) and CD8 T-cell-associated genes (lymphotoxin-β) for some time after clinically effective treatment with etanercept [67,73]. Therefore, these data support the idea that skin resident MCs and autoreactive CD8 T_RM_ cells form localised disease memory with a residual inflammatory signature and their subsequent pathological activation causes tissue-specific inflammation, functioning in both chronic lesions and site-specific recurrence of diseases [49,65,67]. 

**Table 1:** Characteristics of skin T_RM_ and recirculating CD8 T cells.

Characteristic		T_RM_ Cells	Recirculating Cells	Ref.
Trafficking property		Skin-resident	Skin-transient	[10]
Percentage (%) of CD8 T cells in healthy human skin	Epidermis	25%	<5%	[51]
	Dermis	13%	<5%	
Surface markers in skin		CD103^+/−^CD69^+^CD49a^+/−^	CD69^−^	[58]
Requirement for persistency in skin		TGF-β, IL-15/IL-7	TGF-β, IL-15	[56,74,75]
Effector function		CD103^+^ > CD103^−^	Limited	[51,76]
Proliferative capacity		CD103^−^ > CD103^+^	T_CM_ > T_EM_	
Antiviral protection		Critical for secondary infection	Required for optimal response	[77]

Abbreviations: T_RM_ cells, tissue memory T cells; TGF-β, transforming growth factor-β; IL, interleukin; T_CM_, central memory T cells; T_EM_, effector memory T cells.

### 2.3. Modalities of Mast Cell-CD8 T Cell Interplay in the Skin

An increasing number of studies in human skin and mouse models have shown the functional interplay between MCs and CD8 T cells in immune-mediated inflammatory diseases. For instance, increased contacts between MCs and CD8 T cells were detected in tissues affected by T-cell-mediated allergic reactions, AA, rheumatoid arthritis, and multiple sclerosis [33,78,79]. Pioneer studies by Ott and coauthors confirmed the communication between BMMCs and CD8 T cells, demonstrating that MCs activated in an IgE/FcεRI-dependent manner can selectively induce a leukotriene B4 (LTB4)-mediated migration of CD8 T_EM_ cells [80,81]. In recent years, other MC-derived products (e.g., TNF-α, CCL2, CCL3, and CCL5) have been discovered to recruit T cells to inflamed sites in different experimental models [82,83,84]. Furthermore, MCs have been shown to modulate CD8 T cell activities by promoting antigen-specific cytotoxic responses in an experimental autoimmune encephalitis mouse model as well as their activation and cytokine production [85,86,87]. The latter is further supported by the in vitro co-culture study by Stelekati et al. which showed that the physical interaction between MCs and CD8 T cells induces the release of soluble mediators, such as IL-2 and IFN-γ [80]. MC-derived TNF-α is also shown to enhance CD8 T cell proliferation as well as IFN-γ expression in the in vitro co-culture setting [88]. Moreover, activated MCs enhance the cytotoxic and exocytosis potential of CD8 T cells, as measured by the expression of granzyme B and lysosomal-associated membrane protein-1 (LAMP-1) [89]. 

While these data indicate that MCs have a broad regulatory effect on CD8 T cell functions, the literature reports that activated CD8 T cells affect MCs activities. Stelekati et al. discovered that activated CD8 T cells upregulate the expression of MHC-1 and costimulatory molecule 4-1BB on MCs in an in vitro co-culture system, although whether this is due to T-cell-derived chemokines or cell–cell interaction needs further investigation [80]. T-cell-secreted cytokines/chemokine products, such as β-chemokines (e.g., MIP-1α and MCP-1), have been reported to directly induce mouse MC degranulation, while human MCs are unresponsive [90]. 

Therefore, the modulation of activities between MCs and CD8 T cells is bidirectional. However, the mechanism implicated in such crosstalk and how it is altered in pathological conditions is largely unknown. Here, we summarise the current knowledge, which is limited, on MC-CD8 T cell crosstalk and indicate different means of interaction, in human skin: cell–cell contact (MHC-TCR, ligand–receptor binding) and soluble mediators (chemokine/cytokine).

## 3. Modalities of Mast Cell–CD8 T Cell Interactions in the Skin: Cell–Cell Contact

### 3.1. MHC-TCR Mediated Mast Cell–CD8 T Cell Interactions

Direct cell–cell contact is vital to intercellular communication in the immune system [91]. In vitro co-culture studies have proved the need for direct cell contact for antigen-specific MC-driven CD8 T cell activation that was inhibited by a trans-well membrane system allowing soluble mediator diffusion [80,84]. To date, formal demonstration of a direct cellular contact/mechanistic link between MCs and CD8 T cells in human skin remains open. 

**Figure 1:**
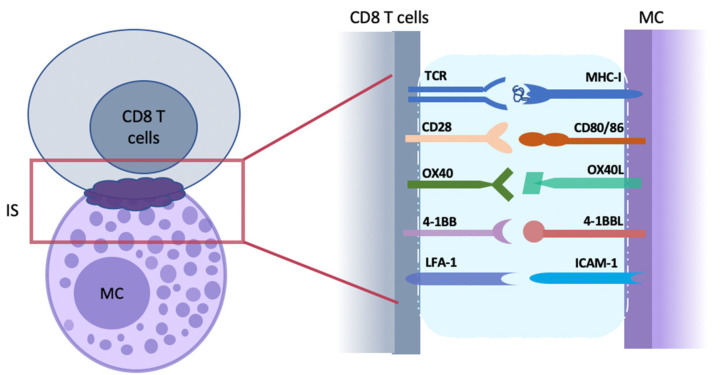
Direct cell–cell interactions between mast cells (MCs) and CD8 T cells. The tight apposition of antigen-presenting MC and antigen-recognizing CD8 T cell initiates the formation of an immunological synapse (IS; purple cloud) at the contact interface. IS is a platform with a nano-scale gap/distance formed for cell–cell communication and continent exchange. As the first step of T cell activation, the antigenic peptides bind with MHC-I and are presented to CD8 T cells that recognize them with TCR on the cell surface. Following with MHC-TCR interaction at IS, other molecules required for enhanced T-cell activation and structure modulation are recruited. Costimulatory molecules pairs (such as CD28 and CD80/86, OX40 and OX40L, and 4-1BB and 4-1BBL) provide a second signal for the activation of naïve CD8 T cells. ICAM-1/IFA-1 are adhesion molecular pairs that are important for the formation and strengthening of IS structure. MC, mast cells; IS, immunological synapse.

The area of direct contact between MCs and effector cells restricted by adherens junctions (AJ) is called an immunological synapse (IS) and contains structures for cell communication and material exchange (Figure 1) [92,93,94]. MCs can process and directly present antigens to effector or memory CD8 T cells via peptide-MHC (pMHC) class I complexes, and to CD4 T cells via pMHC class II complexes [95]. The dynamic TCR-MHC synapse structure of facilitates the recognition of APC-presented antigens by T cell receptors (TCR). Meanwhile, the persistent movement of plasma membranes and the crucial change of biochemical contents, including the polarised exposure of granule-stored mediators, such as CXCL8 and TNF-α, play a key role in the initial steps of T cell activation [96]. 

Although MCs are known to present antigens to T cells in vitro, as discussed in more detail by Katsoulis-Dimitriou et al., it appears that their antigen-presenting function is setting-sensitive, especially for naïve T cells [97,98]. MCs, therefore, are qualified as nonprofessional APCs [99]. Orinska et al. reported that activated BMMCs express IFN-β, which is known to augment MHC-I expression on APCs and synergise with chemokines to enhance CD8 T cell activation and proliferation [100,101]. This finding was supported by Ogasawara et al., who reported that CD8 T cells lacking IFN-β signalling components are hypo-responsive to antigen stimulation with ovalbumin (OVA) in vitro [101]. To date, how pMHC-TCR binding at the T cell/APC surface triggers and sustains TCR signalling is not fully explained. In addition to antigen presentation, MHC-I molecules can also reversely induce signal transduction in multiple cell types, including MCs, but the precise role of reverse MHC class I signalling in MCs, specifically in CD8 T cell activation in vivo, remains elusive [102,103]. 

**Figure 2:**
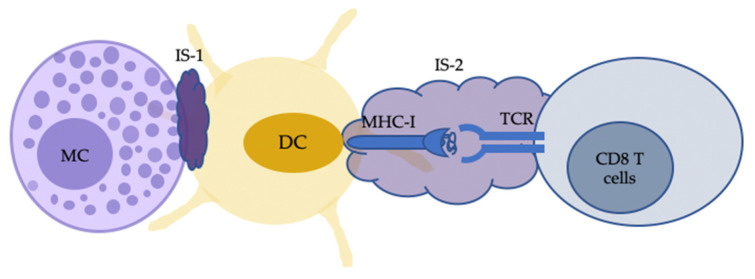
Indirect MC-CD8 T cell interactions can also occur with the antigen cross-presentation by dendritic cells (DCs). Activated MCs, following IgE–FcεRI crosslinking with antigen, can trigger the formation of an immunological synapse (IS) with immature DCs (IS-1). The IS facilitates the transfer of MC-internalised antigens from MCs to DCs. DCs then process and present the transferred material to CD8 T cells with IS-2 for cell activation. MC, mast cells.

Communication between MCs and CD8 T cells can also be facilitated by other immune cells. MHC-I-dependent CD8 T cell activation is not solely the result of MC antigen-presentation. Carrol-Portillo et al. showed that activated MCs can trigger the formation of cellular/immunological cognate interaction with DC that can modulate T cell activation (Figure 2) [104]. This is supported by the impaired MHC class I-dependent CD8 T cell priming due to the decreased recruitment of DCs in MC-deficient mice with the parasitic skin infection by Leishmania major [105].

In addition, the intracellular molecular machinery, such as cytoskeletal forces, have been taken into consideration on the basis of the receptor deformation model, which initiates TCR signaling by mechanical stress [106,107]. 

Although little is known about the nature of the IS between MC and CD8 T cells, the MC-CD4 T cell synapse shown to be generated by OVA-specific murine MCs, primed with IFN-γ and IL-4, may provide some clues. Gaudenzio et al. visualised the synapses where a fraction of MCs function as APC and present antigen in the context of MHC class II to CD4 T cells, leading to a cell–cell contact-dependent MC activation and polarised IFN-γ expression by CD4 T cells [108]. T cell subsets exhibit heterogeneity in the spatial structure and dynamic of IS organisation, which is linked to the type of antigens, the nature of APC, and the activation status of T cells [109].

Cell adhesion molecules have also been discussed in cell signalling and communication via IS [110,111]. The direct human MC-T cell contact is dependent on adhesion molecules and has been shown to be partially mediated by intracellular adhesion receptor 1 (ICAM-1)/lymphocyte function-associated antigen-1 (LFA-1) ligation, as shown in the in vitro study undertaken by Lundequist et al. [27]. The interaction among ICAM-1, adhesion receptor expressed on MCs, and LFA-1 on T cells contributes to the structural stabilization of IS [111]. In addition, ICAM^+^ MCs release exosomes that induce T cell proliferation and cytokine production. However, the ICAM-LFA axis is less studied in MC-CD8 T cell interaction, and the colocalisation is weak in the study by Bertolini and colleagues [33].

### 3.2. Costimulatory Molecule-Mediated Mast Cell–CD8 T Cell Interactions

In addition to MHC-TCR binding, effective T cell activation requires costimulatory receptor signals that result from the engagement of TNF receptor superfamilies (TNFRSFs) and ligands, which have been well-documented to elicit distinct functional heterogeneity in regulating MC-mediated T cell activation (Figure 1) [112,113]. MCs express various costimulatory molecules, including TNF/R family members (e.g., OX40L, 41-BB, and CD153) whose expression is increased in an autocrine manner by TNF-α [84]. Blockade of costimulatory signals or engagement of inhibitory costimulatory pathways, such as CTLA-4 and PD-1, results in tolerance and suppression of allogeneic T cell responses [114]. Therefore, the costimulatory signalling via these axes has been well-discussed in CD8 T cell responses (Table 2) [115,116]. 

#### 3.2.1. CD28-CD80/CD86 Ligation

CD28, a member of the immunoglobulin (Ig) superfamily, is constitutively expressed on naïve T cells (50% in human and 100% in mouse CD8 T cells) [120]. The ligands CD80 (B7-1) and CD86 (B7-2) are expressed at high levels on APCs, including MCs and DCs, following maturation [121]. The CD28-CD80/86 pathway provides essential costimulatory signals for naïve T cell activation and optimal CD8 T cell responses to a variety of pathogens, including influenza and vesicular stomatitis virus [113,122]. The CD28-CD80/CD86 binding complex initiates costimulatory signal transduction cascades dependent on cytoplasmic tail motifs on CD28 [120]. The interactions lead to the recruitment of downstream signalling proteins, including phosphatidylinositol-3-kinase (PI3K), growth factor receptor-bound protein 2 (Grb2), and tyrosine kinase Itk, followed by the activation of protein kinase B (PKB/Akt) and classical nuclear factor–κB (NF-κB) signalling pathway [120,123]. CD28 ligation is also regulated by guanine-nucleated exchange factor Vav1 and filamina A [124]. 

CD28 ligation on T cells functions as an amplifier of TCR signalling [125]. This, in turn, increases T cell sensitivity and specificity to TCR stimulation, which is associated with enhanced membrane raft clustering and IS stabilisation [125,126]. In particular, CD28 ligation plays an amendatory role in priming the CD8 T cell response when TCR ligation is impaired, though other costimulatory pathways, such as 4-1BB ligation, can substitute CD28 engagement [113]. For example, in the absence of CD28, 4-1BB stimulation restores CD8 T cell responses to influenza viral infection [127]. CD28-CD80/CD86 signalling enhances production of IL-2, which provides weak signalling for a primary CD8 T cell response to acute infection but strongly programmes memory cell differentiation during recall response [120,128]. In line with this, Williams et al. reported that IL-2Ra deficiency does not significantly affect the primary antiviral T cell response, whilst impairment of the secondary response was observed [128]. Fuse et al. used the mixed chimaera mouse model against DNA virus infection and found IL-2 can restore the CD8 T cell recall response to infection in the absence of CD28 costimulation [121]. Moreover, CD28 ligation can enhance T cell survival, in part by upregulating the expression of anti-apoptotic proteins, including Bcl-XL [129]. 

In addition, the contribution of the CD28-CD80/CD86 to antigen-specific CD8 T cell responses has been supported by CD28 blockade and studies in CD28-deficient mice [128]. Selective blockade of CD28 signalling, either with anti-CD80/CD86 or cytotoxic lymphocyte-associated antigen-4 (CTLA4)-Ig (soluble protein binding to CD80/86), has been applied clinically in the treatment of autoimmune diseases, such as rheumatoid arthritis, and in melanoma [130,131]. 

#### 3.2.2. CD28-CD80/86 Ligation in Psoriasis

Psoriasis is a chronic, non-contagious immune-mediated skin disorder affecting 60 million people worldwide [132]. Plaque psoriasis, the most common form accounting for 90% of cases, presents as well-circumscribed, red/grey plaques covered with silvery scales, most commonly located on extensor surfaces of limbs and on the scalp, and either remains localised or can affect any skin surface [133,134]. Histopathologically, psoriasis has three principal features: abnormal keratinocyte proliferation, dilated blood vessels, and a dermal inflammatory infiltration of immune cells, including macrophages, MCs, neutrophils, and lymphocytes [133]. It is well established from research on human and mouse models that the TNF-α-IL-23-IL-17 axis plays a central role in the pathogenesis of psoriasis [135,136]. In accordance with this, FDA-approved biologic agents targeting this axis have shown high efficacy for psoriasis treatment [137,138]. Analysis of biopsy samples has identified MC degranulation as an early and constant morphological change in psoriasis [139]. In addition, CD8 T cells have been suggested to drive the development, flares, and chronicity of psoriasis, with an increase in numbers found in psoriasis lesions, as previously discussed [49]. In psoriasis, the expression of the CD28-CD80/86 axis is significantly higher in diseased compared with normal skin, suggesting a critical role for the costimulatory signal via this axis in the pathogenesis of the disease (Table 2) [117]. CD28 costimulation has been studied in the development and maintenance of psoriasis lesions, with disease improvement and decreased skin-infiltrating T cells observed in patients treated with CD28 blockers [140]. However, the CD28-CD80/86 axis does not solely provide costimulatory signals for resident T cell activation, which explains the recurrence of psoriasis in the presence of CD28-CD80/86 blockade [141]. 

#### 3.2.3. CD28-CD80/CD86 Ligation in Mast-Cell-Mediated CD8 T Cell Activation

In addition to playing a role in MC-CD8 T cell interactions, CD28-CD80/CD86 is reported to modulate DC activities which, in turn, regulate CD8 T cell activation [86]. In a murine lymphocytic choriomeningitis virus (LCMV) model, the absence of MCs correlates with an impairment of DC activities, with a decrease in number as well as in CD80 and CD86 expression [86]. Despite the vital role of CD28 ligation for CD8 T cell function and survival, CD28^+^ CD8 T cells are a small population of highly differentiated CD8 memory T cells derived from PBMCs, with increased expression of CD57 and the adhesion molecule CD2 [129]. The relatively low numbers of CD28^+^ T cells in homeostatic conditions has led to the hypothesis that these cells are residential cells that have undergone previous antigen exposure and are related with aging and weakened immunity [129]. 

#### 3.2.4. OX40-OX40L

Aside from CD28 ligation, OX40-OX40L is a well-studied costimulatory pathway that provides a second signal for T cell activation. OX40 (CD134, TNFRSF4), a type 1 transmembrane molecule of TNFRSF, is primarily expressed on activated CD8 and CD4 T cells, but not on naïve T cells [142]. OX40L (CD252, TNFSF4), the ligand of OX40, is expressed by a wide range of activated cells, including MCs and NK cells [84]. Immunohistochemical analysis by Kotani et al. showed that the majority of MCs in human skin express OX40L, while CD4 and CD8 T cells expressing OX40 are found in the epidermis and dermo-epidermal junction zone [143]. In addition, Kashiwakura et al. reported that certain MC populations, such as tonsillar MCs, showed significantly higher expression of OX40L and 4-1BBL (a CD8 T-cell-specific costimulatory molecule) than lung MCs. Furthermore, FcεRI aggregation led to an overall increase of OX40L and 4-1BBL on MCs [112]. The expression of OX40L on human MCs and OX40 on CD8 T cells is increased by MC-derived soluble TNF following MHC-dependent MC-mediated T cell activation [84,142,144]. OX40-X40L signalling is delayed and appears relevant only 2~3 days after initial antigenic stimulation. This seems to be partially due to the fact that a high level of OX40 expression is secondary to CD28-CD80 and CD86 ligation and IL-2-IL-2R signalling [142,145]. OX40-OX40L signalling augments T cell proliferation, effector T cell survival, and memory cell differentiation; however, CD4 T cells are more dependent on this axis than CD8 memory T cells [116]. Nakae et al. reported that the OX40-OX40L signalling pathway modulates MC-mediated IgE/Ag/FcεRI-dependent T cell proliferation [84]. Bansal-Pakala et al. reported that antibody blocking of OX40 not only significantly inhibited CD8 T cell expansion in response to OVA-mediated activation, but was followed by considerable CD8 T cell death [146]. Similarly, in a T cell–MC coculture system, Ag-stimulated T cell proliferation, as well as sequent cytokine expression (IL-17 and IFN-γ), was significantly reduced by anti-OX40L monoclonal antibody [84]. This can be explained by the regulatory role of OX40 signals on cytokine expression of CD4 T cells [147]. 

#### 3.2.5. OX40-OX40 Ligation in Atopic Dermatitis 

Atopic dermatitis, or eczema, is a chronic pruritic skin condition affecting children and adults, with an overall prevalence of 2.7%~20.1% and 2.1%~4.9%, respectively [148,149]. The symptoms include persistent pruritus and localised or disseminated eczematous lesions. AD has two forms: allergic and nonallergic form, with the former consistently associated with IgE-related allergic reactions against environmental allergens in a disease sequence referred to the “atopic march” [150,151]. The key components in AD pathogenesis are epidermal barrier disruption/dysfunction and associated cutaneous immune dysregulation, which exhibits a Th2-cytokine profile (e.g., IL-4 and IL-13) [152]. MCs have been found not only to be in higher numbers but to increase cytokine production in diseased skin in AD models and AD patients [153,154]. The MRGPRX2-dependent MC activation and the rapid release of pre-stored product tryptase is linked to itch, a hallmark of AD [37]. Similarly, CD8 T cells are found abundant in the epidermis and dermis of human AD skin lesion and show a distinct cytokine expression profile compared to psoriasis [53]. 

The increase in number of OX40L^+^ MCs in lesional skin, especially those in direct contact with CD8 T cells, has been reported in autoimmune skin diseases (Table 2) [33,155]. Immunofluorescence analysis of skin biopsies has shown the colocalisation of OX40L^+^ and OX40^+^ cells in lesional skin, indicating that the OX40-OX40L axis, which is vital for shaping the Th2 memory cell pool, and directly involved in the pathogenesis of AD [118]. The serum level of OX40 is decreased, while its expression on CLA^+^ skin-homing T cells is enhanced, indicating increased migration of pathogenic T cells to inflamed skin. The strategy of blocking OX40-OX40L interaction for the treatment of AD has shown promise [156]. For example, KHK4083, an anti-OX40 monoclonal antibody, resulted in sustained improvement of AD as measured by the Eczema Area and Severity Index (EASI) in a Japanese study [157]. These data support the pathogenic involvement of MC-T cells via OX40-OX40L system in the skin of AD.

### 3.3. Soluble Mediators in Mast Cell–CD8 T Cell Interactions

**Figure 3:**
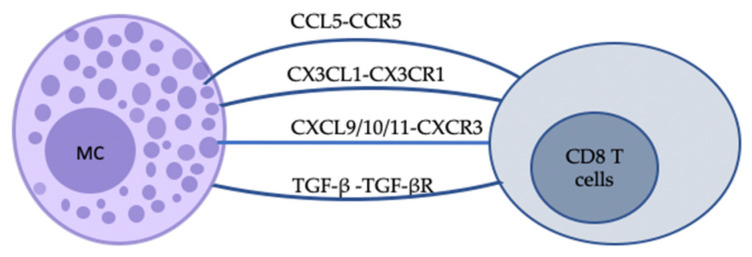
Schematic representation of some of the indirect bidirectional interactions between MCs and CD8 T cells. The communication between MCs and CD8 T cells can be realised by soluble mediators. Chemokine–chemokine ligand interactions can provide instructive signals for CD8 T cells. For example, CCL5-CCR5 and CXCL9/10/11-CXCR3 binding can promote cell migration to Th1-type inflammatory sites, while the latter also plays a role in cell activation and differentiation. TGF-β-TGF-βR signals can promote CD8 T cell residency in skin.

In addition to direct cell–cell contact, cytokines and chemokines expressed by activated MCs are known to alter the migratory and functional properties of T cells, as summarised in Figure 3. Elevated levels of chemokines, such as CCL5 and chemokine ligands (CXCL10, CXCL9, and CXCL11), have been confirmed in blood and skin samples from patients with skin disease, and these may serve as clinical biomarkers for clinical severity [158,159,160,161]. CCL5 binds to CC chemokine receptor 5 (CCR5), expressed on memory or effector type CD8 T cells, while CCR5 has also been associated with CCL3- or CCL4-sensing [162,163]. Orinska et al. reported the increased expression of CCL5 in BMMCs activated by poly I:C in a toll-like receptor 3 (TLR3)-dependent and degranulation-independent manner, which shape the chemotactic property of activated MCs for CD8 T cells [100]. McAlpine et al. further classified the selective recruitment of memory CD8 T cells toward MCs after viral stimulation, which can be inhibited by a CCR5 antagonist [83,164]. Compared with two other CXCR3 ligands, CXCL9 and CXCL11, the importance of the CXCR3-CXCL10 pathway has been emphasised with its role in the recruitment of CD8 T_RM_ cells into the epidermis in mouse models [165,166]. Early studies have shown that the expression of chemokine genes of the CXC family is regulated by IFN-α/β signalling [101]. Further study on LCMV-infected mice has demonstrated that the ligand-mediated recruitment and activity of CXCR3-expressing CD8 T cells with central memory type can be initiated by IFN-γ [167].

Owing to its dual properties of chemoattractant (soluble form) and cellular adhesive compound (membrane-bound form) depending on its state, CX3CL1 is unique among chemokines released by activated endothelial cells as well as immune cells, such as MCs [168]. CX3CL1 binds to a single receptor, CX3CR1, which is expressed on a variety of cells, including effector CD8 T cells, NK cells, and monocytes [169]. To note, CX3CR1 is expressed on effector type of CD8 memory T cells (T_EM_) compared with those possessing proliferative capacity (T_CM_) [170,171]. The involvement of the CX3CL1-CX3CR1 axis in T cell homing to the CX3CL1-producing cells in skin as well as their correlation with disease severity is supported by studies investigating normal and inflamed tissues and/or blood from human and mouse models of AD and psoriasis [172,173,174,175,176]. In addition, TGF-β, one of the chemoattractants released by MCs, can upregulate CD103 expression on CD8 T_RM_ cells via TGF-β-TGF-βR signals and initiate adhesive interactions, thereby contributing to the formation and longevity of CD8 T_RM_ cells in skin [177].

#### Mediators Signalling in Vitiligo

Chemokine and cytokine networks have been well-studied in a broad range of inflammatory skin disorders. Vitiligo is a chronic depigmenting skin disorder with an estimated prevalence of 1% in the general population, with the majority of cases developing before the age of 30 [178]. It is characterised by chalky-white, well-demarcated macules and patches resulting from the selective loss of functional melanocytes [179]. Research into the pathogenesis of vitiligo has indicated several mechanisms involved in the progressive destruction of melanocytes, including genetic, metabolic abnormalities, autoimmune responses, and oxidative stress [180]. The progression of vitiligo is driven by IFN-γ-producing autoreactive CD8 T cells which leads to the Th1-specific destruction of melanocytes. The IFN-γ-chemokine signalling axis is responsible for autoreactive CD8 T cell recruitment and localisation as well as their effector function in the inflamed site through a positive feedback loop [181]. The role of MCs in vitiligo pathogenesis might be both positive and negative [182]. Histopathological studies have revealed an increased number of MCs as well as their degranulation in the lesional dermis of vitiligo [182]. In an in vitro culture system, MC-derived histamine stimulates melanogenesis via histamine H2 receptors in melanocytes through a process involving protein kinase A activation and induces persistent hyper-melanosis in response to the excessive expression of paracrine factors (e.g., SCF) by epidermal keratinocytes [183,184]. In lesional skin and serum from vitiligo patients, the gene expressions of CXCL10 and CXCR3 are significantly upregulated [185]. In line with this finding, higher frequencies of circulating CXCR3^+^CD8 T cells and skin-infiltrating CXCR3^+^ T cells were reported in patients with progressive vitiligo compared with those with stable disease and in healthy controls [161]. Furthermore, increased serum levels of CXCL10 were associated with the clinical severity of vitiligo and decreased with disease stabilisation in response to effective treatment. Blockade of the CXCL10-CXCR3 axis via CXCL10-knockout or CXCL10-neutralising antibody in mouse models has been shown to halt the progression of vitiligo and induce re-pigmentation [45,186]. To this end, CXCL10-CXCR3 axis may play a vital role (such as T cell recruitment to inflamed sites) in vitiligo pathogenesis and provide new therapeutic strategies. Similar to vitiligo, analysis of skin samples from psoriasis patients shows that CXCR3 is vital for CD8 T cell trafficking to the affected dermis and then into the epidermis in psoriasis plaques [187]. 

## 4. Conclusions

MCs and CD8 T cells play vital roles as effector and/or immunoregulatory cells in the skin immune system. There is evidence that these cells and their mediators are involved in the pathogenesis of several skin inflammatory diseases as well as their reoccurrence. The crosstalk between immune cells is a relatively unexplored research avenue in skin disorders but offers considerable promise as a potential approach to novel therapeutic strategies. It is apparent that functional MC-CD8 T cell associations exist under both physiological and pathological conditions. The concept that MC-CD8 T cell interactions contribute to CD8 T-cell-mediated diseases dates back years but has still not been systematically followed up, partially due to the tissue-resident nature of MCs in the skin and the lack of proper disease models [188]. A growing body of evidence describes that CD8 T cell activation requires three signals: TCR ligation, costimulation, and cytokine signalling. Pathways, including OX40-OX40L and CD28-CD80/CD86, have been found to play an important role in skin disorders and to be involved at different stages of CD8 T cell activation/differentiation but as yet have not been linked to MC-CD8 T cell interactions. In the future, the elucidation of the mechanisms and molecules that tightly regulate MC-CD8 T cell interaction as well as their alterations in different skin diseases remains a crucial goal.

## Figures and Tables

**Table 2:** Costimulatory molecules and their ligands in skin disorders.

CD8 T Cell Receptors	MC Ligands	Receptor Family	Disease	Ref.
CD28	CD80 (B7-1); CD86 (B7-2)	IgG-CD28/B7	psoriasis	[117]
OX40 (CD134)	OX40L (CD252)	TNF/TNFR	atopic dermatitis; alopecia areata	[33,118]
4-1BB (CD137)	4-1BBL	TNF/TNFR	psoriasis	[119]

Abbreviations: MC, mast cells; Ig, immunoglobulin; TNF, tumour necrosis factor; TNFR, tumour necrosis factor receptor.

## Data Availability

Not applicable.

## Abbreviations

AA	alopecia areata
AD	atopic dermatitis
APC	antigen-presenting cells
AJ	adherens junctions
BMMC	bone-marrow-derived mast cells
CLA	cutaneous lymphocyte-associated antigen
CCL	CC chemokine ligand
CCR	CC chemokine receptor
CHS	cutaneous contact hypersensitivity
CTLA	cutaneous lymphocyte-associated antigen
CRC	colorectal cancer
CXCR	C-X-C chemokine receptor
CXCL	C-X-C chemokine ligand
CLT	cytotoxic lymphocyte-associated antigen-4
DAMP	damage/danger-associated molecular pattern
DC	dendritic cells
EASI	Eczema Area and Severity Index
FcεRI	high-affinity IgE Fc receptor
GBD	Global Burden of Disease
Grb2	growth factor receptor-bound protein 2
GWAS	genome-wide association studies
IgICAM-1	immunoglobulinintracellular adhesion receptor 1
IS	immunological synapse
IL	interleukin
LAMP-1	lysosomal-associated membrane protein-1
LCMV	lymphocytic choriomeningitis virus
LFA-1	lymphocyte function-associated antigen-1
LTB4	leukotriene B4
MAPK	mitogen-activated protein kinase
MC	mast cell
MHC	major histocompatibility complex
NF-κB	nuclear factor-κB
NK	natural killer
OVA	ovalbumin
PGD2	prostaglandin D2
PI3K	phosphatidylinositol-3-kinase
PKB/Akt	protein kinase B
pMHC	peptide-MHC
PUVA	psoralen and ultraviolet light A
RAF	rapidly accelerated fibrosarcoma
RAS	rat sarcoma
S1PR1	sphingosine-1-phosphate receptor 1
SCF	stem cell factor
VEGF	vascular endothelial growth factor
TGF-β	transforming growth factor-β
TLR3	toll-like receptor 3
TNF	tumour necrosis factor
TNFR	tumour necrosis factor receptor
TNRSF	TNF receptor superfamilies
TNSF	TNF superfamilies
T_CM_	central memory T cells
T_EM_	effector memory T cells
T_RM_	resident memory T cells
